# Supplementary material for: Method for Isolation of Myxozoan Proliferative Stages from Fish at High Yield and Purity: An Essential Prerequisite for In Vitro, In Vivo and Genomics-Based Research Developments
Source: Cells. 2022 Jan 23;11(3):377. doi: 10.3390/cells11030377 (PMC8833907; doi:10.3390/cells11030377)
Supplement: Supplementary file 1 [file cells-11-00377-s001.zip › cells-1500300-supplementary.pdf]

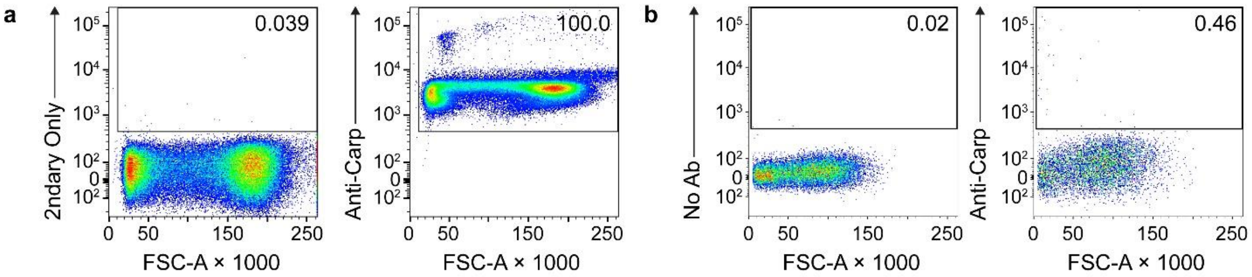

Figure S1. Validation of the polyclonal mouse anti-pan-carp cell antibody. (**a**) Labelling of almost 100% of circulating carp blood erythrocytes and leukocytes in non-infected SPF fish, at high fluorescence intensity (right graph), in comparison to the control where only the secondary antibody was applied (left graph). This particular trial was based on using unconjugated mouse anti-pan-carp cell antibody (labelled 'Anti-Carp'). (**b**) Testing of the APC/R-PE-conjugated Anti-Carp on purified parasite to exclude non-specific binding that would interfere with distinguishing parasite from host cells. Contamination by cross-linkage is minor (0.46% of cells wrongly identified as host cells, right graph) when compared to the negative control (left graph) and staining intensity of carp leukocytes in (**a**). Axes represent fluorescence intensity of antibody staining (Y-axes) and cell size (X-axes, forward scatter area [FSC-A]).

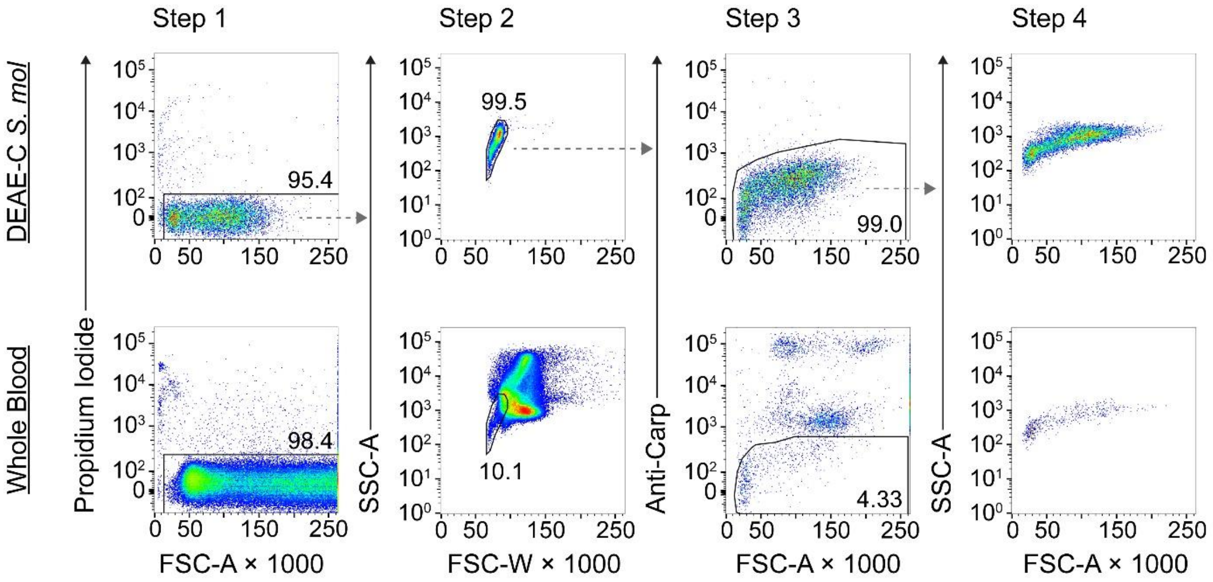

Figure S2. Stepwise optimization of gating strategy during flow cytometric analyses to ensure accurate distinction between parasite and host cells. Top row representing DEAE-C-purified *S. molnari* BS and bottom row representing infected host blood. Step 1: propidium iodine staining (Y-axes showing intensity) to determine the number of dead cells (percentage of live cells is given in graphs) and exclude them from downstream analyses. Step 2: side scatter area (SSC-A; complexity/granulosity) versus forward scatter width (FSC-W; cell size) showing physical profile of live cells. In this particular blood sample (bottom row), 90% of cells can be readily excluded because they do not match the morphological profile of DEAE-C-purified BS. However, in the remaining 10%, carp blood cells are still among parasites. Step 3: use of polyclonal 'Anti-Carp' antibody (Y-axis showing intensity) to further exclude the non-stained parasite population by low fluorescence intensity (bottom row) and for comparison with the population of DEAE-C-purified BS (top row). In this case, 4.33% of cells are identified as BS in the blood sample. Step 4: Overlap of physical parameters (size and complexity) of the parasite population identified in fish blood by antibody labelling (bottom row) with that of pure DEAE-C-isolated BS (top row). Performing these comparisons ensures that the events we are ultimately analyzing (Step 4) are not host cells wrongly identified as parasite cells.

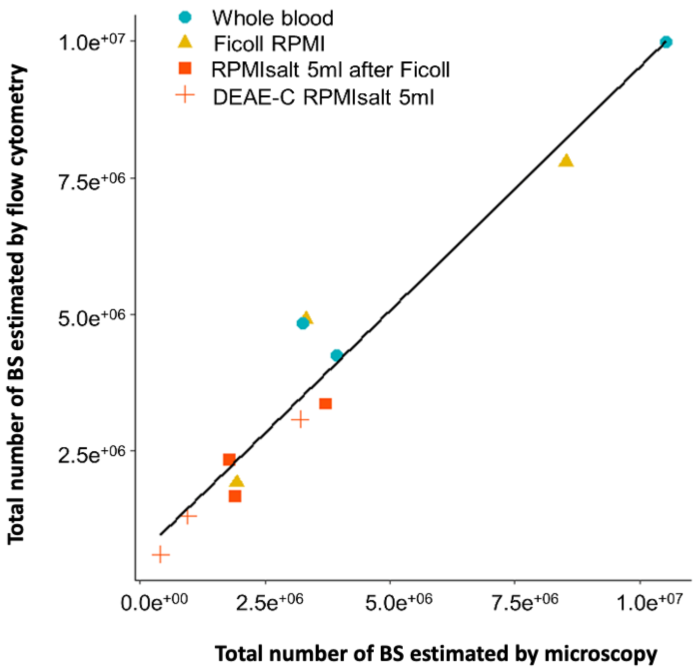

Figure S3: Correlation between *S. molnari* BS numbers determined by microscopy and flow cytometry, before and after different isolation steps and procedures.
